# Sentinel lymph node biopsy mapped with methylene blue dye alone in patients with breast cancer: A systematic review and meta-analysis

**DOI:** 10.1371/journal.pone.0204364

**Published:** 2018-09-20

**Authors:** Jiyu Li, Xiao Chen, Ming Qi, Yanshuang Li

**Affiliations:** 1 Department of Breast and Thyroid Surgery, Shandong Provincial Hospital Affiliated to Shandong University, Jinan, Shandong Province, China; 2 Department of Neurology, Jinan Central Hospital Affiliated to Shandong University, Jinan, Shandong Province, China; University of Campinas, BRAZIL

## Abstract

**Background:**

Methylene blue dye is easy to obtain in developing countries and can be used in sentinel lymph node mapping for breast cancer. However, the accuracy of methylene blue alone for sentinel lymph node mapping in breast cancer has not been well defined. In this study, we collected data to assess the feasibility and accuracy of sentinel lymph node biopsy mapped with methylene blue alone in patients with breast cancer.

**Methods:**

We searched the PubMed, EMBASE, and Cochrane Library databases from January 1, 1993, to March 31, 2018. Selected studies had to have a defined group of patients with breast cancer in which MBD alone was used as the mapping technique for SNB.

**Results:**

18 studies were included in this study. The combined identification rate was 91% [95% confidence interval (CI): 88%-94%, I^2^ = 68.3%], and the false negative rate was 13% (95% CI: 9%-18%, I^2^ = 36.7%). The pooled sensitivity, negative predictive value, and accuracy rate were 87% (95% CI: 82%-91%, I^2^ = 37.5%), 91% (95% CI: 87%-93%, I^2^ = 32.4%) and 94% (95% CI: 92%-96%, I^2^ = 29%), respectively.

**Conclusions:**

This meta-analysis found that mapping sentinel lymph node locations with methylene blue dye alone results in an acceptable identification rate but an excessive false negative rate according to the American Society of Breast Surgeons’ recommendations. Caution is warranted when using methylene blue dye alone as the mapping method for sentinel lymph node biopsy.

## Introduction

Sentinel lymph node biopsy (SNB) was first reported in cutaneous melanoma by Morton et al. in the early 1990s [1]. The sentinel lymph node (SN) concept was soon adopted for use in breast cancer patients[2] and led to significant improvement in the management of the axilla in breast cancer surgery. Currently, SNB has become a standard procedure for axillary staging in early breast cancer [3–5]. As a minimally invasive surgery, SNB can accurately stage the axilla and leads to less morbidity than axillary lymph node dissection (ALND) [6–8].

The mapping method is one of the most important factors affecting the identification rate (IR) and false negative rate (FNR) of SNB in breast cancer. Giuliano conducted intraoperative lymphatic mapping and identified the SN using only blue dye[2]. Krag investigated the use of radioisotopes for SN identification [9], while Albertini was the first to identify the SN using a combination of blue dye and radioisotope techniques[10]. Several studies have reported that the combined use of blue dye and radioisotopes is significantly superior to blue dye alone for SNs identification [11–13]. Although there is no standard mapping technique for SNB, the combination of blue dye and radioisotope techniques is thought to be more reliable and is currently the most widely used method for SNB mapping in breast cancer.

Unfortunately, many hospitals in developing countries, including China, do not currently have the ability or qualifications to provide nuclear medicine and equipment. Although the radiation exposure during SNB using radioisotopes is limited and is safe for pregnant surgeons and patients [14–16]. Concern about the hazards of radiation exposure is also an obstacle for the use of the combined method. Furthermore, in these countries, there is limited access to patent blue and isosulfan blue. Therefore, MBD alone is sometimes used to map SN localization in these countries.

MBD is cheaper than patent blue or isosulfan blue and is easier to obtain in developing countries. Simon first reported that MBD could serve as an alternative to isosulfan blue in combination with radioisotopes for SNB in breast cancer[17], and similar conclusions were drawn by other researchers[18–21]. Recently, several studies reported that blue dye alone was sufficient for identifying SNs in breast cancer [22–24]. Thus, the use of MBD alone as a mapping method for SNB seems feasible and may expand the use of SNB in developing countries. Although several studies have used MBD alone to map SNs in breast cancer, the patient selection criteria and details of the mapping methods varied across individual studies. Thus, we performed the present meta-analysis to collect data to assess the feasibility and accuracy of SNB mapped with MBD alone in patients with breast cancer.

## Materials and methods

### Literature search strategy

**Fig 1:**
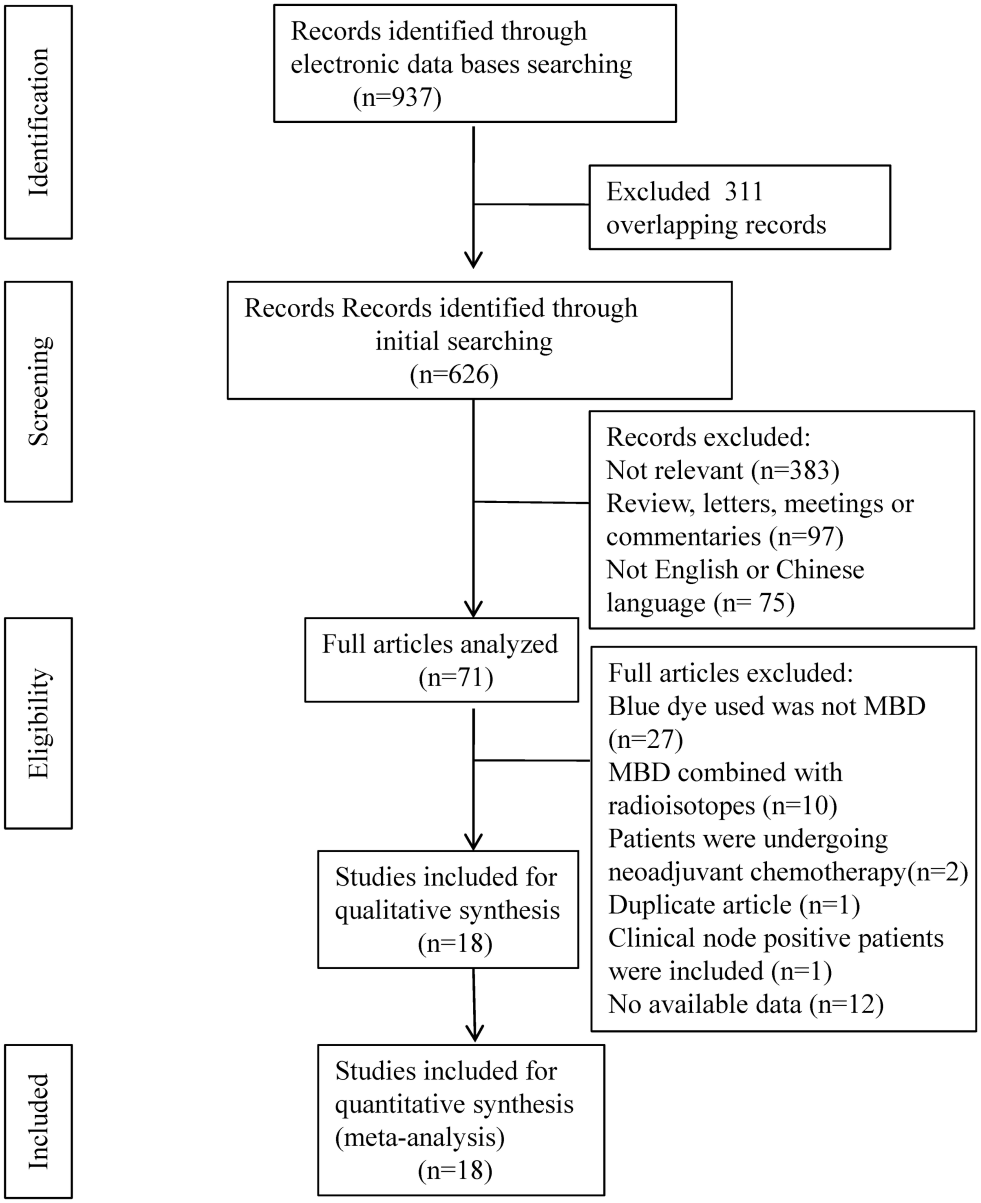
Flow diagram of the literature search and individual studies identified for this systematic review and meta-analysis.

We searched the PubMed, EMBASE, and Cochrane Library databases from January 1, 1993, to March 31, 2018. The following medical subject heading (Mesh) terms were used: ‘breast cancer’, ‘sentinel lymph node biopsy’, and ‘blue dye’. Furthermore, we used combinations of ‘breast cancer’, ‘sentinel lymph node biopsy’ and ‘blue dye’ as free text terms. The references of selected articles were also reviewed to identify additional relevant articles. Articles published in English and Chinese were selected. Letters, editorials, case reports and reviews were excluded from the study. The search strategy is presented in Fig 1.

### Study inclusion criteria

Selected studies had to have a defined group of patients with breast cancer in which MBD alone was used as the mapping technique for SNB. The included studies described the IR and/or FNR of SNB. Studies that used other blue dyes, such as patent blue or MBD, combined with radioisotopes were excluded from this meta-analysis. Patients receiving neoadjuvant chemotherapy were excluded. Studies that included clinical node-positive patients for SNB were excluded. For studies with overlapping study populations, only the most recent study with the most inclusive number of patients was included.

### Study quality assessment

QUADAS 2, a standardized tool for the quality assessment of diagnostic accuracy studies, was adapted for this review [25]. QUADAS 2 comprises four domains for assessing the risk of bias: patient selection, index testing, reference standards, and flow and timing. If the answers to all signaling questions of a domain are "yes," then the risk of bias to the corresponding domain can be considered low. If any answer is "no", then the risk of bias to the domain can be considered high. Applicability concerns were judged using similar criteria. All studies were independently analyzed by two authors. The questions adopted in our review are listed in S1 File.

### Data extraction

Data were extracted by 2 independent reviewers, and the accuracy of the data was verified by 2 other reviewers. Disagreements were resolved by consensus after discussion. Abstracted information regarding study characteristics included first author, publication year, study origin, number of patients, age of patients, tumor size, clinical axillary node status, intra operative evaluation of SN, MBD concentration, MBD dose, injection site and number of SNs harvested.

To evaluate the performance, the number of true positives and false negatives results were extracted.

### Statistical analysis

The meta-analysis in this study was conducted using R version 3.2.2 for Windows (R: A language and environment for statistical computing; R Foundation for Statistical Computing, Vienna, Austria, http://www.R-project.org/).

The IR for SNB was defined as the number of patients with successfully identified SNs divided by the total number of patients for whom SNB was attempted. The results of each successfully identified SN were further categorized as true positive (TP), true negative (TN), or false negative (FN). Four test performance parameters were evaluated: sensitivity [TP/(TP+FN)], FNR [FN/ (FN+TP)], NPV [TN/(TN+FN)], and AR [(TP+TN)/total number of successful SNB].

The meta-analysis of IR, FNR, accuracy rate (AR), negative predictive rate (NPV) and SNB sensitivity was conducted using the metaprop function in the R-meta package. Individual studies were weighted by study size and by the inverse of the variance of individual point estimates. The heterogeneity of the studies was evaluated using the inconsistency statistic (I^2^) [26]. For outcome measures without significant heterogeneity among studies (P > 0.10), proportions were calculated using a fixed-effect model; otherwise, a random-effect model was employed. Publication bias was displayed graphically using funnel plots. The effect of MBD dose and injection site on the IR and FNR was determined using the chi-squared test. Two-sided P-values < 0.05 were considered significant.

## Results

### Characteristics of the included studies

A total of 1,559 patients in 18 studies that met the inclusion criteria were analyzed in our meta-analysis [27–44]. Our search strategy is presented in Fig 1. The 18 studies were published between 2000 and 2017. Five studies were from China, 2 were from Turkey, and 1 study each was from Chinese Taipei, Egypt, Italy, the United States, the United Kingdom, Jamaica, Greece, India, Serbia, Pakistan, and Indonesia. All of the studies included a group of patients for which MBD alone was used as the mapping technique for SNB. All patients in all 18 studies were clinical axillary node negative. Four studies used touch imprint cytology and 3 used frozen section analysis for the intraoperative evaluation of SNs. Ten studies used 1% MBD, and 1 study used 2% MBD. Three studies used 2 ml MBD, and 7 studies used 5 ml MBD. Peritumoral MBD injection for SNB was used in 8 studies, and subareolar MBD injection was used in 6 studies. The characteristics of the 18 studies, including patient age, tumor size, clinical axillary node status, intraoperative evaluation of SN, MBD concentration, MBD dose and injection site are listed in Table 1.

**Table 1:** Characteristics of the included studies.

Study	Publication year	Origin	No. of patients	Age (years)	Tumor size	Clinical axillary node status	Intra operative evaluation of SN	Concentration of MBD	Dose of MBD (ml)	Injection site	No. of SN
Su et al.	2000	China	52	28–70	T_1-3_	N_0_	ND	2%	2	Peritumoral	1–15
Yu et al.	2002	Chinese Taipei	221	26–82	<3cm	N_0_	TIC	NR	5	Peritumoral	NR
Chen et al.	2002	China	24	34–85	≤3.5cm	N_0_	ND	1%	2–4	Peritumoral	NR
Nour et al.	2004	Egypt	54	32–65	2-5cm	N_0_	ND	NR	5	Subareolar	1–4
Tang et al.	2005	China	38	29–65	T_1-2_	N_0_	ND	1%	2	Peritumoral	NR
D'Eredita	2006	Italy	40	40–78	T_1-2_	N_0_	ND	NR	4	Subareolar	1–8
Golshan et al.	2006	USA	141	29–82	0–5.3cm	N_0_	TIC or FSA	1%	5	Subareolar	1–9
Varghese et al.	2007	UK	173	58.3*	1.52*cm	N_0_	ND	1%	1	Subareolar	1–4
Huang et al.	2007	China	89	26–80	NR	N_0_	TIC	NR	2	Subareolar	NR
East et al.	2009	Jamaica	24	NR	T_1-2_	N_0_	ND	1%	5	Subareolar	1–2
Kaklamanos et al.	2011	Greece	126	57.8*	1.76*cm	N_0_	FSA	1%	5	Subareolar or Peritumoral	1–4
Ge et al.	2011	China	51	28–73	NR	N_0_	ND	1%	4–6	Subareolar or Peritumoral	NR
Khanna et al.	2011	India	102	31–67	T_1-3_	N_0_	TIC	1%	5	Peritumoral	1–2
Coskun et al.	2012	Turkey	53	NR	NR	N_0_	ND	1%	10	Subdermal and Subareolar	NR
Özdemir et al.	2013	Turkey	32	25–82	NR	N_0_	ND	1%	5	Peritumoral	1–2
Djruisic	2014	Serbia	152	33–82	0.1–4.8cm	N_0_	FSA	NR	0.2,0.5or 1.0	Subareolar or Peritumoral	1–4
Bakhtiar et al.	2016	Pakistan	81	23–70	T_1-3_	N_0_	ND	1%	3–5	Peritumoral	NR.
Brahma et al.	2017	Indonesia	96	25–69	1-10cm	N_0_	ND	1%	5	Subareolar or Peritumoral	1–8

* Mean value.

NR: not recorded, ND: not done, TIC: touch imprint cytology, FSA: frozen section analysis.

### IR of SNB

**Fig 2:**
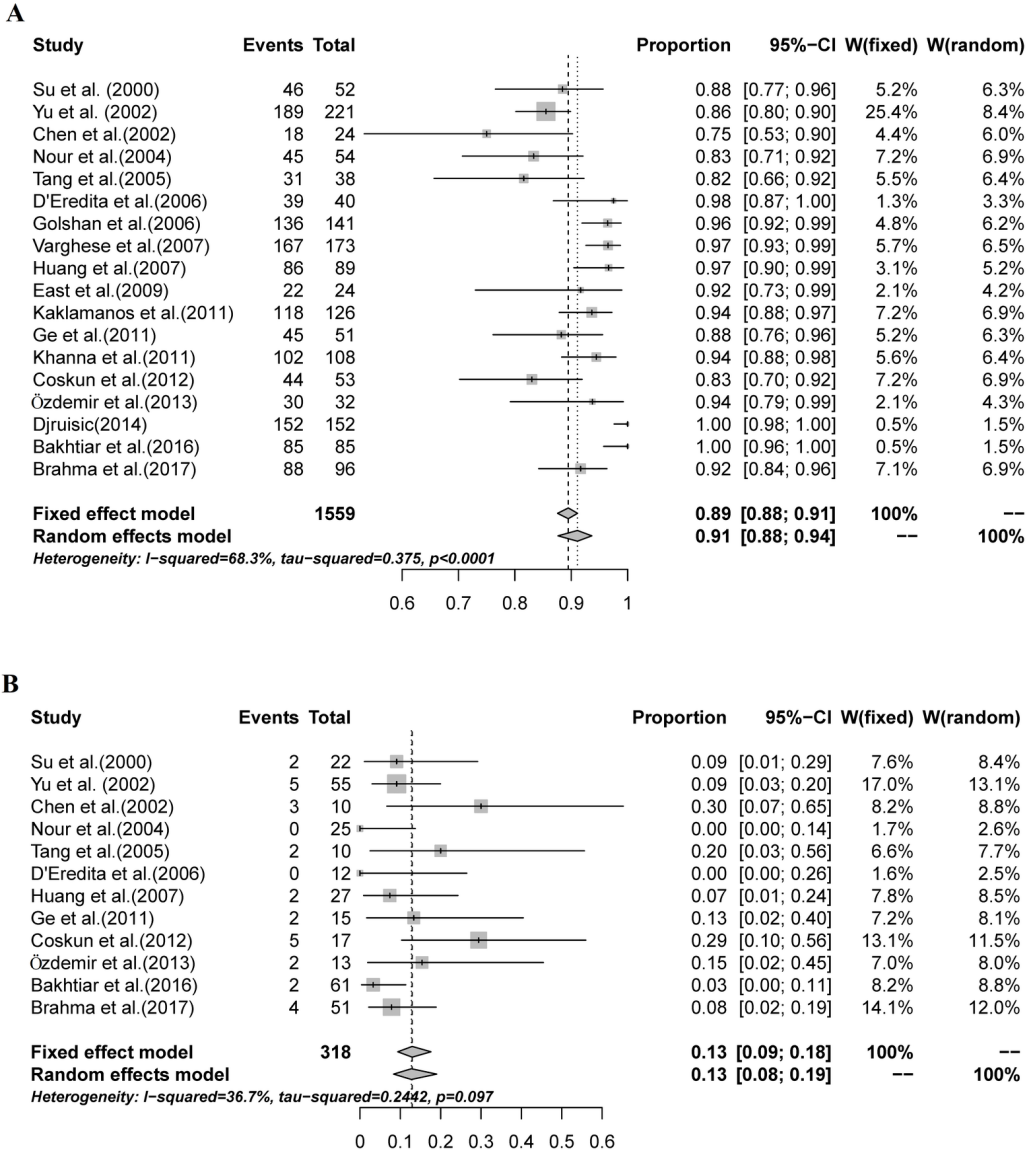
The combined IR and FNR of SNBs mapped with MBD alone. A: A random-effects model was used to estimate the combined IR, with a result of 91% (95% CI: 88%-94%, I^2^ = 68.3%); B: A fixed-effects model was used to estimate the pooled FNR, with a result of 13% (95% CI: 9%-18%, I^2^ = 36.7%).

All 18 studies provided data for the analysis of IR. The IR of SNB in individual studies ranged from 75%-100%. The I^2^ value was 68.3%, reflecting a high degree of IR heterogeneity among the included studies. Therefore, a random-effects model was used to estimate the combined IR, with a result of 91% [95% confidence interval (CI): 88%-94%; Fig 2A).

### FNR of SNB

Four studies provided no data for the analysis of FNR. In the remaining 12 studies, the FNR ranged from 3%-30%. Minimal FNR heterogeneity was found among the studies (I^2^ = 36.7%; Fig 2B). A fixed-effects model was used to estimate the pooled FNR, with a result of 13% (95% CI: 9%-18%).

### Sensitivity, NPV and AR of SNB

**Fig 3:**
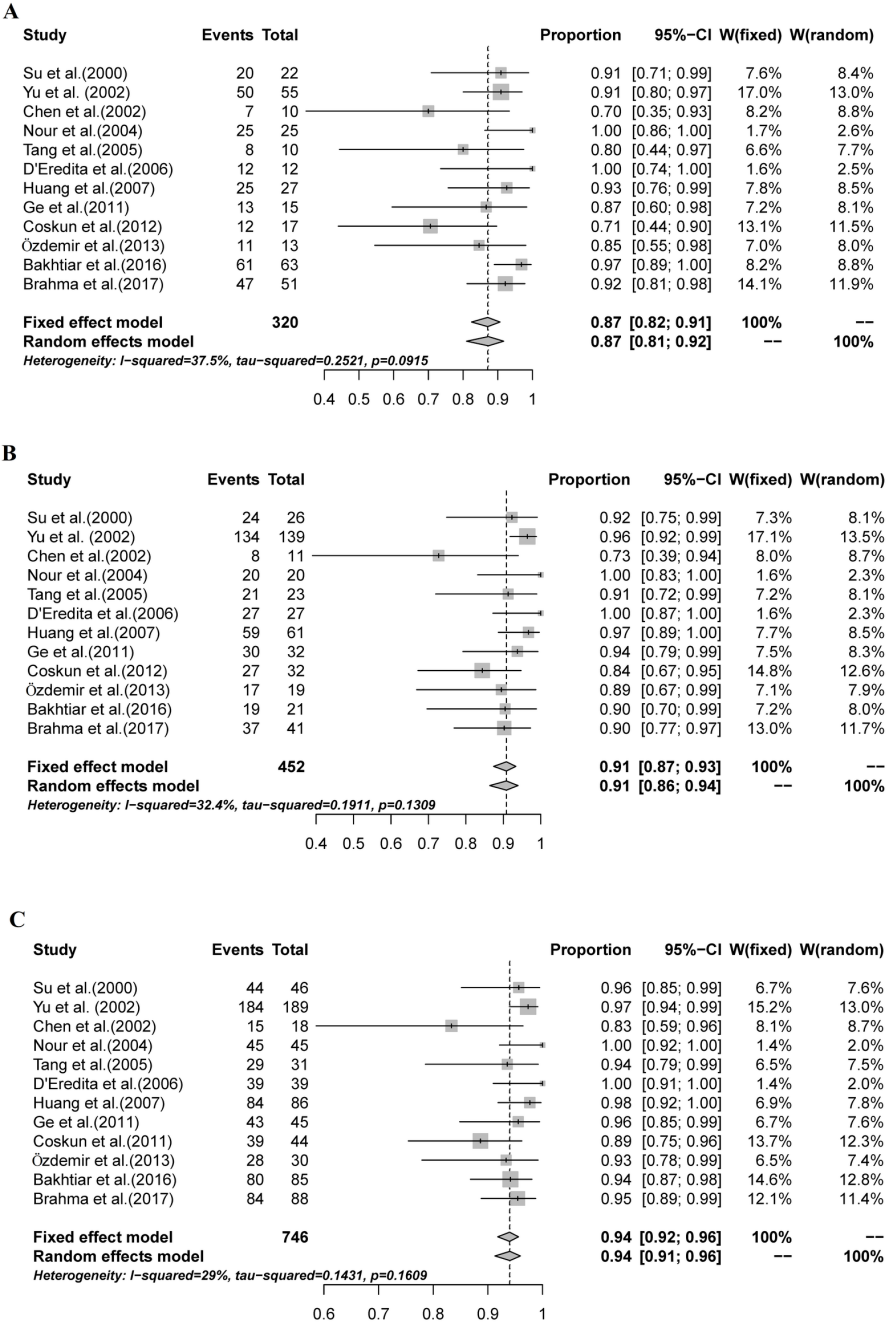
The combined sensitivity, NPV, and AR of SNBs mapped with MBD alone. A: The pooled sensitivity was 87% (95% CI: 82%-91%, I^2^ = 37.5%); B: The combined NPV was 91% (95% CI: 87%-93%, I^2^ = 32.4%); C: The overall AR was 94% (95% CI: 92%-96%, I^2^ = 29%).

Three SNB test performance parameters were analyzed: sensitivity, NPV, and AR. Meta-analyses of these parameters provided a summary sensitivity estimate of 87% (95% CI: 82%-91%, I^2^ = 37.5%; Fig 3A). The summary NPV estimate was 91% (95% CI: 87%-93%, I^2^ = 32.4%; Fig 3B), and the summary AR estimate was 94% (95% CI: 92%-96%, I^2^ = 29%; Fig 3C).

### Comparison of peritumoral and subareolar MBD injection for SNB

There is controversy regarding the optimal injection site of the tracing agent. We compared the use of peritumoral with and subareolar MBD injection in SNB. Peritumoral MBD injection for SNB was used in 8 studies, and subareolar MBD injection was used in 6 studies. In the Kaklamanos study, the patients were randomized into peritumoral or subareolar injection groups. Five other studies using multiple MBD injection sites were excluded from this analysis.

**Fig 4:**
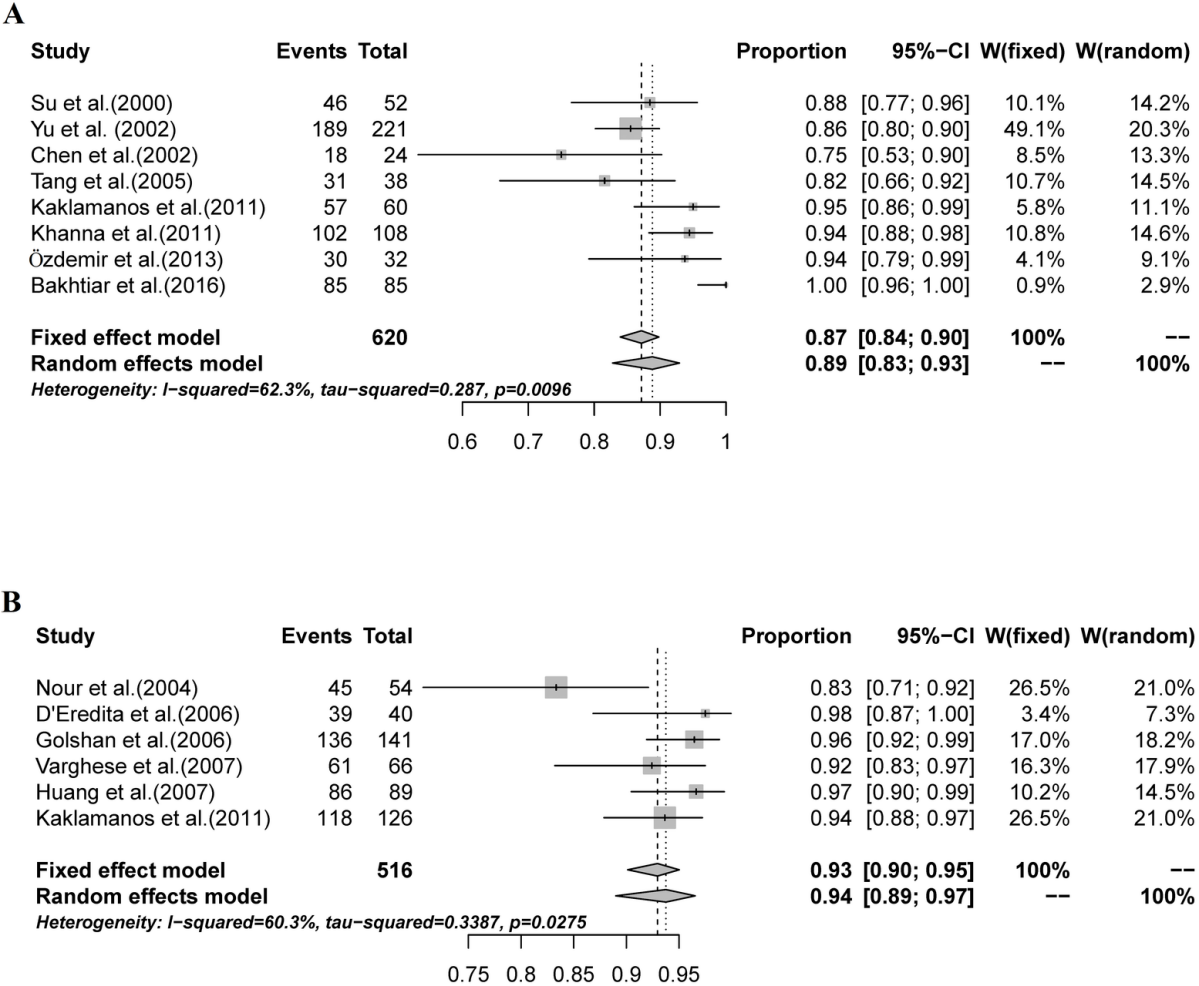
The combined IR for studies using peritumoral injection and studies using subareolar injection. A: The combined IR for studies using peritumoral injection was 89% (95% CI: 83%-93%, I^2^ = 62.3%); B: The combined IR for studies using subareolar injection was 94% (95% CI: 89%-97%, I^2^ = 60.3%).

The pooled IR for studies that used peritumoral injection was 89% (95% CI: 83%-93%, I^2^ = 62.3%; Fig 4A), while in studies using subareolar injection, the pooled IR was 94% (95% CI: 89%-97%, I^2^ = 60.3%; Fig 4B). The IR for SNB in studies using subareolar injection was significantly higher than that in studies using peritumoral injection (P = 0.015, Table 2).

**Table 2:** IR of SNB according to MBD injection site.

MBD Injection site	No. of studies	No. of patients SNB attempts	No. of patients SN successfully identified	IR (95% CI)
Peritumoral	8	620	558	89% (83%-93%)
Subareolar	6	516	485	94% (89%-97%)

**Fig 5:**
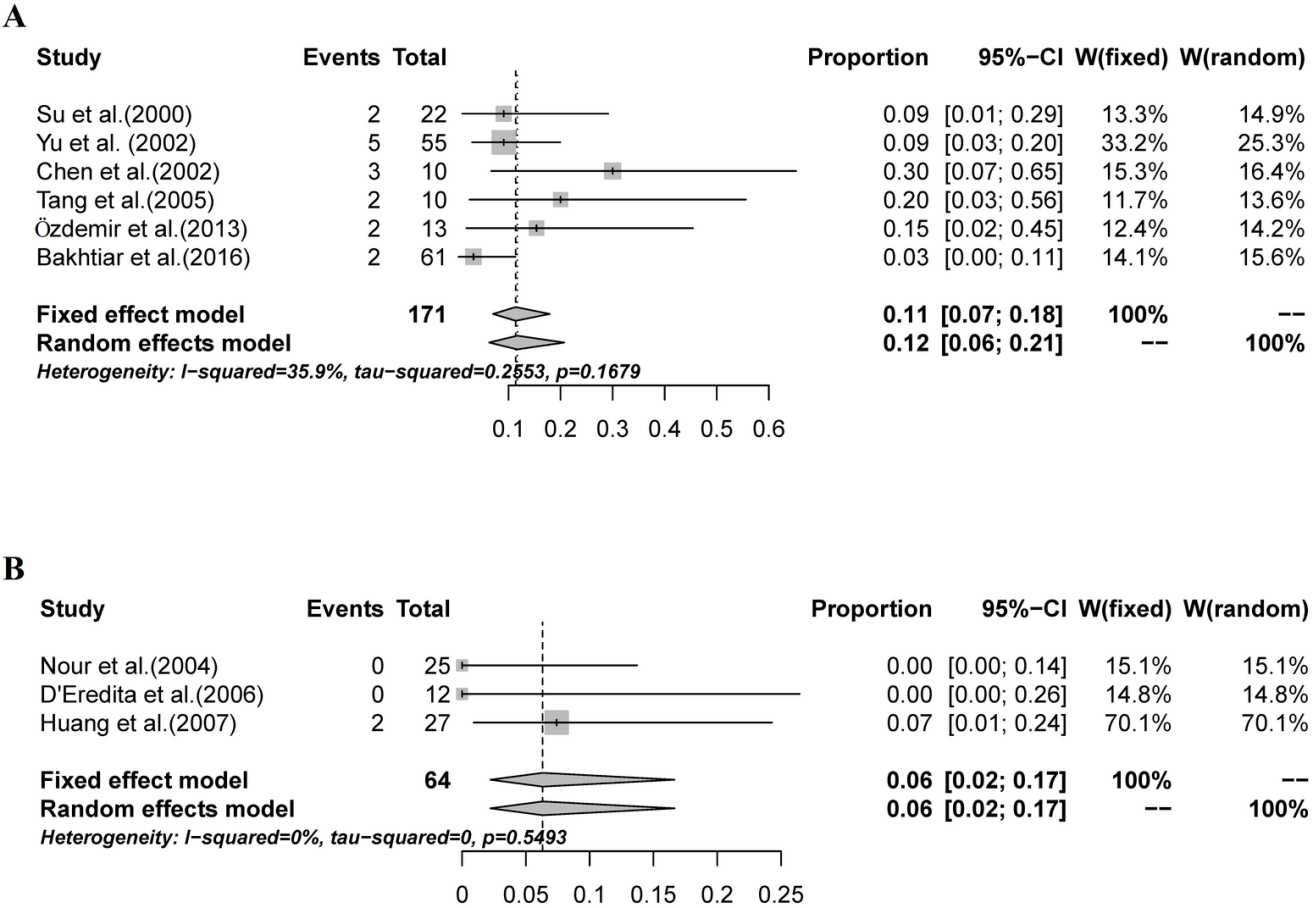
The combined FNR for studies using peritumoral injection and studies using subareolar injection. A: The combined FNR for studies using peritumoral injection was 11% (95% CI: 7%-18%, I^2^ = 35.9%); B: The combined FNR for studies using subareolar injection was 6% (95% CI: 2%-17%, I^2^ = 0%).

The combined FNR of 6 studies using peritumoral injection was 11% (95% CI: 7%-18%, I^2^ = 35.9%; Fig 5A). The combined FNR of 3 studies using subareolar injection was 6% (95% CI: 2%-17%, I^2^ = 0%; Fig 5B). No significant difference in the FNR of SNB was detected between studies using peritumoral and subareolar MBD injection (P = 0.110, Table 3).

**Table 3:** FNR of SNB according to MBD injection site.

MBD Injection site	No. of studies	No. of patients with positive axillary lymph nodes	No. of patients with false negative SNs	FNR (95% CI)
Peritumoral	6	171	16	11% (7%-18%)
Subareolar	3	64	2	6% (2%-17%)

### Comparison of 2 ml and 5 ml of MBD injection for SNB

**Fig 6:**
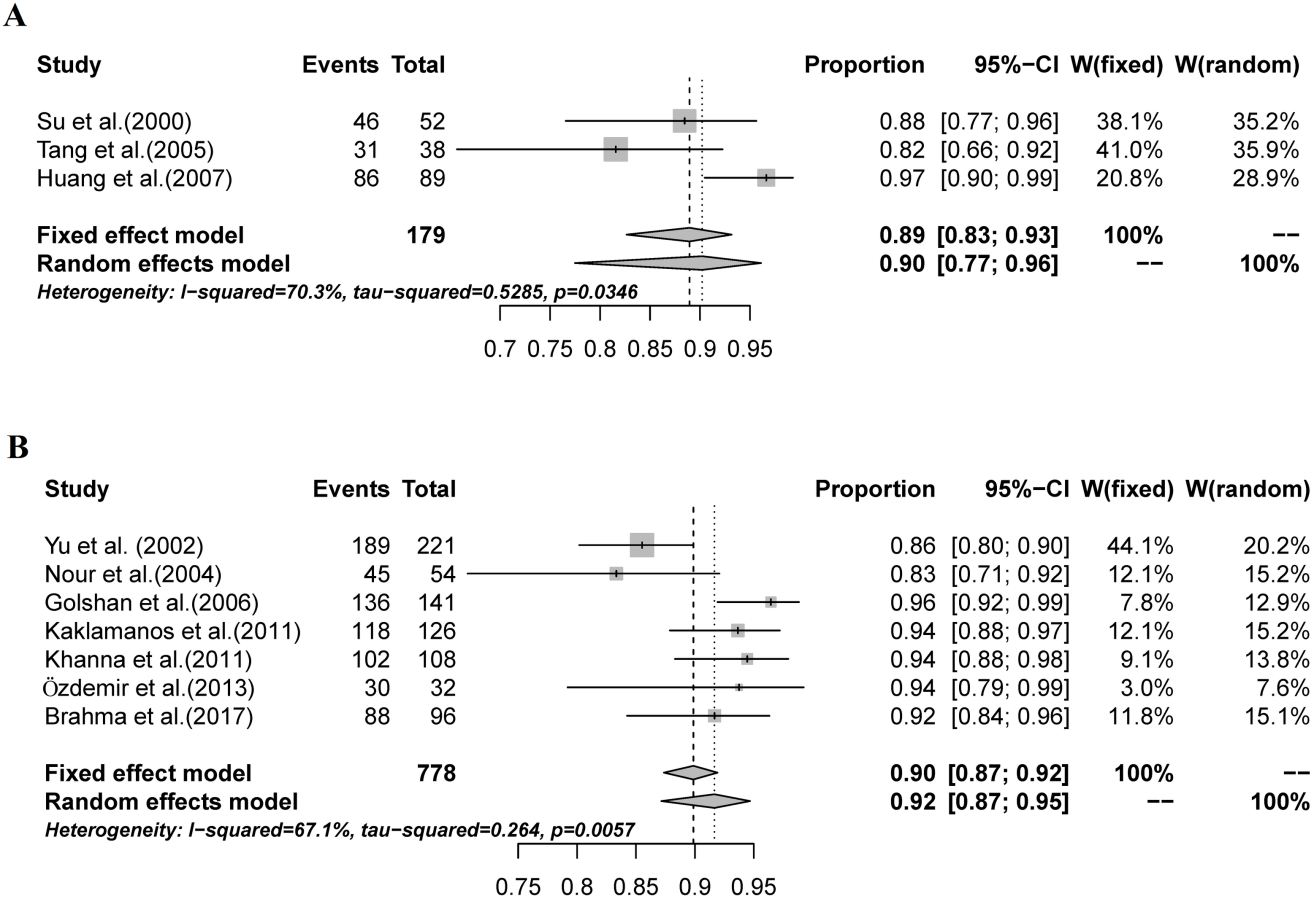
The combined IR of SNB according to different MBD dose. A: The combined IR for the studies that used a 2-ml injection of MBD was 90% (95% CI: 77%-96%, I^2^ = 70.3%); B: The combined IR for the studies that used a 5-ml injection of MBD was 92% (95% CI: 87%-95%, I^2^ = 67.1%).

**Fig 7:**
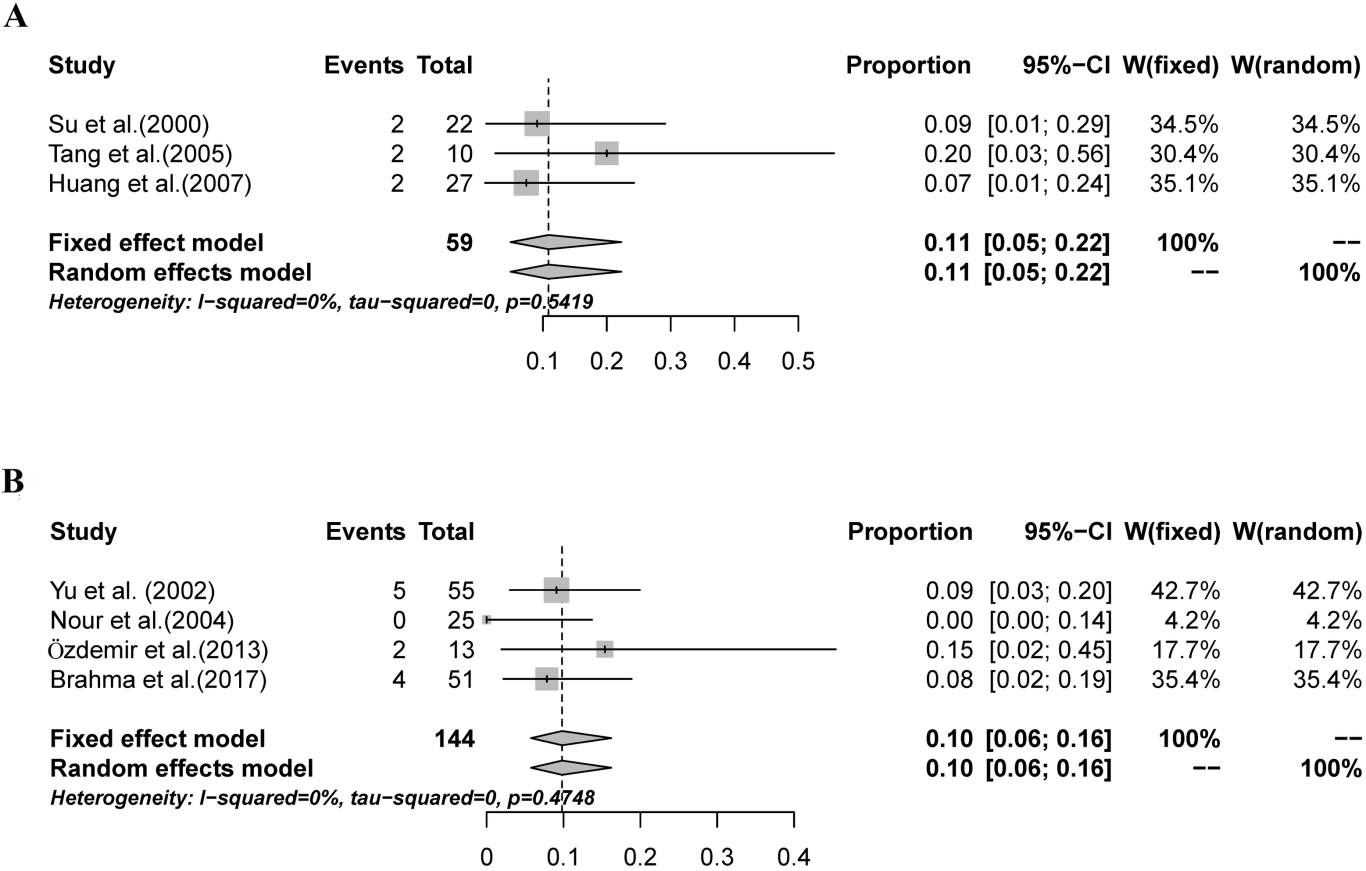
The combined FNR of SNB according to different MBD dose. A: The combined FNR for the studies that used a 2-ml injection of MBD was 11% (95% CI: 5%-22%, I^2^ = 0%); B: The combined FNR for the studies that used a 5-ml injection of MBD was 10% (95% CI: 6%-16%, I^2^ = 0%).

We compared the combined IR and FNR of SNB according to different MBD dose. The combined IR for the studies that used a 2-ml injection of MBD was 90% (95% CI: 77%-96%, I^2^ = 70.3%; Fig 6A); for the studies that used a 5-ml injection of MBD, the combined IR was 92% (95% CI: 87%-95%, I^2^ = 67.1%; Fig 6B). No significant difference was detected between the two groups of studies (P = 0.980, Table 4). The combined FNR for the studies that used a 2-ml injection of MBD was 11% (95% CI: 5%-22%, I^2^ = 0%; Fig 7A), for the studies that used a 5-ml injection of MBD, the FNR was 10% (95% CI: 6%-16%, I^2^ = 0%; Fig 7B). No significant difference in the FNR of SNB was detected between the studies that used 2 ml MBD and those that used 5 ml (P = 0.555, Table 5).

**Table 4:** IR of SNB according to MBD injection dose.

MBD dose	No. of studies	No. of patients SNB attempts	No. of patients SN successfully identified	IR (95% CI)
2ml	3	179	163	90% (77%-96%)
5ml	7	778	708	92% (87%-95%)

**Table 5:** FNR of SNB according to MBD injection dose.

MBD Injection dose	No. of studies	No. of patients with positive axillary lymph nodes	No. of patients with false negative SNs	FNR (95% CI)
2ml	3	53	6	11% (5%-22%)
5ml	4	133	11	10% (6%-16%)

### Quality of the included studies and publication bias

The quality of each study was assessed using QUADAS 2, and the results are listed in Table 6. All the studies has a high risk of patient selection bias, while all other risks were rated as low.

**Table 6:** Results of quality assessment of the included studies according to QUADAS 2.

Study	Risk of bias				Applicability concerns		
	Patient selection	Index test	Reference standard	Flow and timing	Patient selection	Index test	Reference standard
Su et al.	2	1	1	1	1	1	1
Yu et al.	2	1	1	1	1	1	1
Chen et al.	2	1	1	2	1	1	1
Nour et al.	2	1	1	1	1	2	1
Tang et al.	2	1	1	1	2	1	1
D'Eredita	2	1	1	1	2	2	1
Golshan et al.	2	1	1	1	1	2	1
Varghese et al.	2	1	1	1	1	1	1
Huang et al.	2	1	1	2	2	1	1
East et al.	2	1	1	2	1	1	1
Kaklamanos et al.	2	1	1	1	1	1	1
Ge et al.	2	1	1	1	1	2	1
Khanna et al.	2	1	1	2	2	1	1
Coskun et al.	2	1	1	2	1	1	1
Özdemir et al.	2	1	1	1	1	2	1
Djruisic	2	1	1	2	1	1	1
Bakhtiar et al.	2	1	1	1	2	2	1
Brahma et al.	2	1	1	1	1	2	1

1: low risk 2: high risk

**Fig 8:**
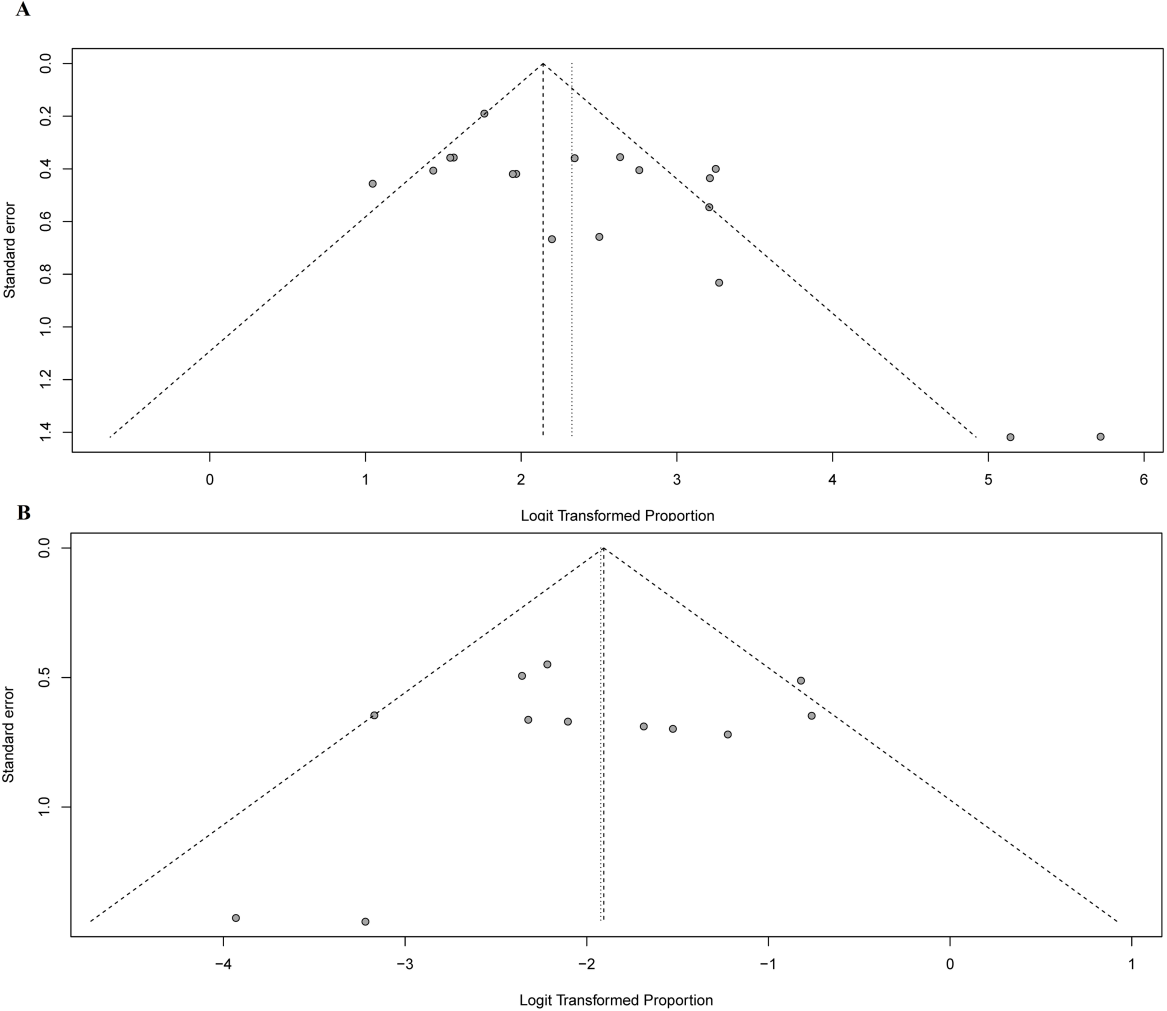
Funnel plots used to assess the effects of publication bias on the IR and FNR. A: Funnel plot to assess publication bias effect on the IR; B: Funnel plot to assess publication bias on the FNR. Each dot represents a separate study. The funnel plots revealed no apparent evidence of publication bias.

To evaluate the publication bias of aggregated data in this meta-analysis, we generated funnel plots for IR and FNR. Overall, the included studies showed good symmetry, suggesting minimal publication bias (Fig 8A & 8B). Begg’s tests for IR and FNR indicated P-values of 0.1204 and 0.8909, respectively. These results confirmed the above conclusions.

## Discussion

The use of blue dye for SNB was first reported in 1992 by Morton and colleagues [1], while Krag et al. introduced the use of radioisotopes for SNB in 1993[9]. To improve the accuracy of SNB, a group from the Moffit Cancer Center recommends the combination of blue dye and radioisotope techniques [10], and several other studies have demonstrated the advantage of this combined method [11–13]. A recent meta-analysis by He et al. concluded that the combination of radioisotope and blue dye in SNB for breast cancer had a higher IR than radioisotope alone[45]. The combination of radioisotope and blue dye is the most widely used technique and is considered the standard technique for localizing SNs. However, radioisotopes are not available at some hospitals, making blue dye alone the only option for localizing SNs.

The most common blue dyes used in SNB are isosulfan blue and patent blue. MBD is also used to map SNs, especially in developing counties with limited access to isosulfan blue or patent blue. MBD is a dark green crystalline compound that becomes dark blue in solution [46]. It is commonly used for diagnostic procedures, such as identifying Barrett’s esophagus[47] and urinary fistulae[48]. Koller first reported the use of MBD injected at the primary tumor site to identify SNs in patients with breast cancer [49]. Several studies have demonstrated that when combined with radioisotopes, MBD can serve as an alternative to isosulfan blue and patent blue for SN mapping[17–21]. Compared with isosulfan and patent blue, MBD is cheaper, easier to obtain in most countries, and has not been associated with potential life-threatening allergic reactions[50]. MBD is even safe for pregnant patients [51]. Hence, MBD may represent a safe and effective alternative to isosulfan and patent blue. However, these blue dyes have different molecular structures, which gives them different characteristics. Isosulfan blue and patent blue show high protein affinity because they contain sulfonic acids, which can combine with the amino groups on the protein surface[52]. In contrast, MBD shows no protein affinity at 37°C [52]. Whether the use of MBD alone is as effective as isosulfan and patent blue for SN mapping in breast cancer requires further clarification.

To our knowledge, this is the first systematic review to provide an overview of the published literature regarding the feasibility and accuracy of SNB mapped with MBD alone in patients with breast cancer.

The 91% IR reported in the present meta-analysis does not differ substantially from the IRs reported in previous studies that mapped SNs using the combination method or other blue dyes alone [22, 53–55]. Pesek’s meta-analysis, which included 183 studies, reported that the overall FNR was 7.5% (95% CI: 7.0–8.1%) when a fixed-effects model was used but dropped to 7.0% (95% CI: 6.1–7.9%) when using a random-effects model was used[56]. Subgroup analysis demonstrated that the FNR was 8.6% (95% CI: 6.7–10.8%) for the dye-only group. The 13% FNR in the current study was higher than that described in Pesek’s report. The IR and FNR are the most important test performance parameters for SNB. To abandon axillary dissection, the American Society of Breast Surgeons recommends an 85% SN IR with an FNR of 5% or less [57]. In patients with breast cancer, the IR for SNB mapped with MBD alone was acceptable, while the FNR was unacceptably high. Han et al. investigated the factors associated with the FNR of SNB in breast cancer[58]and found that a smaller tumor volume, increased number of SNs and increased surgeon experience level were related to a lower FNR. When MBD alone is used as the mapping method for SNB, effective strategies for decreasing FNRs include attempting to identify more SNs, removing any hard or large nodes found adjacent to SNs, selecting an experienced surgeon to perform the procedure, and selecting patients with smaller tumors.

The optimal injection site for mapping tracers remains controversial for SNB in breast cancer. In Mudun’s study, radioisotopes alone were used as the tracing agent to localize SNs, and the IR was superior using intradermal periareolar injection was used compared with peritumoral and subdermal injection [59]. However, in Rodier’s study, the IR was similar in the periareolar and peritumoral injection groups[60]. The IR of 94% for SNB when using subareolar injection was used was significantly higher than the IR of 89% with peritumoral injection (P = 0.003). Ogasawara and his colleagues evaluated lymphatic pathways with indocyanine green fluorescence imaging in patients with breast cancer [61]. In their study, a lymphatic drainage pathway from the periareolar area was detected in 33 out of 37patients, and 12 of these 33 patients had a lymphatic drainage route from the peritumoral area. This anatomic feature of lymphatic drainage of breast helps to explain the higher IR with in subareolar injection compared with peritumoral injection. Thus, to achieve a higher IR when mapping SNs with MBD alone, subareolar injection might be the better choice.

The optimal dose of MBD for SNB is controversial. The most commonly used dose of MBD for SNB are 2 ml and 5 ml. In 18 studies included in current meta-analysis, the volume of MBD varied from 0.1 ml to 10 ml (Table 1). There was no difference in IR or FNR between the studies that used 2-ml versus 5-ml injections of MBD in our meta-analysis. To determine the optimal dose of MBD for SNB, a well-designed study is needed in the future.

Although the use of MBD for SNB in breast cancer has not led to life-threatening allergic reactions, it is not without risk. Stradling and colleagues first reported adverse skin reactions to MBD in patients with breast cancer[62]. For instance, skin, fat and parenchymal necrosis have been reported [63, 64]. Among the 18 studies included in present meta-analysis, Brahma reported that two patients experienced skin necrosis around the MBD injection site[44]; East reported that on patients developed skin and subcutaneous tissue necrosis around the MBD subareolar injection site[36]; and Kaklamanos reported that 3 patients suffered from skin allergic reaction[37]. No fat or parenchymal necrosis was reported. These results demonstrate that MBD is a generally safe blue dye for mapping SNs in breast cancer but that injections into or near the skin should be avoided in patients undergoing breast-conserving surgery.

Only studies published in English or Chinese were included in our meta-analysis, which may have led to publication bias. In addition, studies favoring the use of MBD for SNB in breast cancer are more likely to be published, which may also have contributed to publication bias. However, funnel plots indicated that the presence of publication bias in the present meta-analysis was minimal. Begg’s test further confirmed these results.

In conclusion, based on the findings from this meta-analysis, SNBs mapped with MBD alone result in acceptable IRs of 91% but unacceptable FNRs of 13% according to standards recommended by the American Society of Breast Surgeons. Thus, caution is warranted when using MBD alone as the mapping method for SNB.

## Supporting information

S1 File Questions used to assess the quality of the literature. (DOCX)

S1 Table PRISMA checklist. (DOC)
