# Supplementary material for: Sentinel lymph node biopsy mapped with methylene blue dye alone in patients with breast cancer: A systematic review and meta-analysis
Source: PLoS One. 2018 Sep 20;13(9):e0204364. doi: 10.1371/journal.pone.0204364 (PMC6147575; doi:10.1371/journal.pone.0204364)
Supplement: S1 File — (DOCX) [file pone.0204364.s001.docx]

The following signaling questions were used to assess the quality of the literature:

1 Patient selection

1.1 Risk of bias

1.1.1 Was a consecutive or random sample of patients enrolled?

1.1.2Was a case–control design avoided?

1.1.3 Did the study avoid inappropriate exclusions?

1.2 Applicability concerns

Are there any difference of TMN stage of patients mapped with methylene and other tracer agents?

2 Index test

2.1 Risk of bias

2.1.1 Were the index test results interpreted without knowledge of the results of the reference standard?

2.1.2 If a threshold was used, was it pre-specified?

2.2 Applicability concern

2.2.1 Are there concerns that the index test, its conduct, or its interpretation differ from the review question?

2.2.2 Were patients mapped with raidiocolloid excluded?

3 Reference standard

3.1 Risk of bias

3.1.1 Was the reference standard likely to correctly classify the target conditions?

3.1.2 Were the reference standard results interpreted without knowledge of the results of the index test?

3.2 Applicability concern

Did the reference standard match the question?

4. Flow and timing

4.1 Did all patients receive ALND?

4.2 Was the IR of the study higher than 90%?

4.3 Was the same reference standard used?

4.4 Was there an appropriate interval between the index tests and reference standard?
